# Negative Pressure Wound Therapy as a Bridge to Definitive Reconstruction in Complex Extremity Trauma: A Systematic Review and Meta-Analysis

**DOI:** 10.7759/cureus.108811

**Published:** 2026-05-13

**Authors:** José D Rodríguez Enríquez, Humberto López Gallo, Sarahi Carrillo Barraza, Enrique Córdova López, Eduardo Hernández Ruiz, Maria O Ramirez Miranda, Luis M González colomé, Ricardo E Sotomayor Tuquerrez, Jorge A Pérez Arias, Azucena D Bernal González, Guillermo Padilla Turrubiartes, Paul A Palacios Zaragoza, Miguel Angel Bautista Badal

**Affiliations:** 1 General Surgery, Hospital Regional de Alta Especialidad Dr. Gustavo A. Rovirosa Pérez, Villahermosa, MEX; 2 General Surgery, Hospital Universitario de Torreón, Torreón, MEX; 3 Medicine, Universidad Olmeca, Villahermosa, MEX; 4 General Medicine, Universidad Veracruzana - Campus Xalapa, Xalapa, MEX; 5 General Practice, Universidad Autónoma del Estado De México, Toluca, MEX; 6 General Surgery, Unidad Médica de Alta Especialidad No. 25 Instituto Mexicano del Seguro Social (IMSS), Monterrey, MEX; 7 Medical Physics, Universidad de Colima, Colima, MEX

**Keywords:** bridging treatment, complex extremity trauma, flap failure, negative-pressure wound therapy (npwt), open fracture wounds, soft-tissue reconstruction, surgical site infection

## Abstract

Complex extremity injuries, including open fractures, particularly Gustilo-Anderson type IIIB and IIIC injuries, continue to be a major challenge for all types of institutions and levels of healthcare, a surgical challenge even in specialized trauma centers or hospitals with trained staff characterized by extensive soft tissue defects, bone exposure, and high rates of infection, flap failure, and secondary amputation. Finding the best strategy to reduce morbidity and complications of these injuries remains a challenge for plastic surgery. Negative pressure wound therapy (NPWT) has played an important role as a temporary "bridging" strategy between initial surgical debridement and definitive soft tissue reconstruction. This article is a systematic review and meta-analysis that evaluates the efficacy and safety of NPWT as a bridging therapy before definitive soft-tissue reconstruction in adult patients with complex extremity injuries, compared with conventional dressings or immediate reconstructive procedures.

A systematic review and meta-analysis were conducted in accordance with the Preferred Reporting Items for Systematic Reviews and Meta-Analyses (PRISMA) guidelines. They were prospectively registered with PROSPERO(CRD420261359962), aimed to evaluate and synthesize evidence from comparative studies published between 2009 and 2025. The risk of bias was assessed using the Cochrane Risk of Bias 2 tool for randomized controlled trials (RCTs) and the Newcastle-Ottawa scale for observational studies. The certainty of the evidence was assessed using the GRADE approach.

The evidence reveals an interesting comparison between large, multicenter RCTs and smaller, single-center studies. The two highest-quality trials, the Wound Management of Open Lower Limb Fractures (WOLLF) RCT (n=460) and the Wound Healing in Surgery for Trauma (WHiST) RCT (n=1548), found no statistically significant reduction in rates of deep surgical site infections. The Cochrane review, which combined four RCTs (596 participants), found an uncertain reduction in the risk of infection (RR 0.48, 95% CI 0.20-1.13; very low-certainty evidence). In contrast, multiple smaller RCTs from single-center, resource-limited settings reported statistically significant reductions in acute wound infection (7.5-10% with NPWT vs 25-42% with conventional dressings). A meta-analysis of 10 RCTs (n=2780) found a significant reduction in the overall risk of infection (pooled MD 0.70, 95% CI 0.54-0.90, p=0.005). NPWT consistently allowed for a lower reconstructive ladder, replacing the free flap with skin graft coverage in multiple series, and retrospective data suggest a possible reduction in flap failure rates (6% vs 11%). NPWT as a bridging therapy for complex extremity trauma offers context-dependent benefits. The greatest benefit is expected for Gustilo IIIB/IIIC injuries in resource-limited settings with high baseline contamination and limited reconstructive capacity. In specialized trauma centers with sufficient resources and early debridement, standard wound management achieves equivalent results at a lower cost and with a lower incidence of adverse events. NPWT-d should be considered a superior alternative when available. High-quality, randomized clinical trials specifically designed to evaluate NPWT as a bridging strategy to flap surgery are needed.

## Introduction and background

Complex extremity trauma and open fractures represent one of the most challenging scenarios for surgeons and orthoplastic surgery, combining skeletal instability, severe soft tissue loss, periosteal stripping, and contaminated wound beds, which are major challenges in extremities, making open fractures one of the most difficult cases for orthoplastic surgeons. The Gustilo-Anderson classification, originally described in 1976 and refined in 1984, continues to be the most common type of injury severity grading system, and Type IIIB fractures (extensive periosteal stripping, exposed bone, and soft tissue defects requiring flap reconstruction) and Type IIIC fractures (arterial injury that also requires vascular repair) carry the greatest potential morbidity of infections and limb loss [1,2]. In particular, treating severe open fractures requires urgent surgical debridement, skeletal stabilization, and definitive soft tissue coverage. Historical evidence from Godina’s 1986 series established the '72-hour rule' as a benchmark, demonstrating that microsurgical reconstruction within this window significantly lowered flap failure and infection rates compared to delayed coverage, thus establishing the '72-hour rule' that was the de facto best practice in reconstructive surgery for decades [2,3]. Nevertheless, there is rarely any realistic opportunity for early definitive closure, especially among polytrauma victims who require physiologic optimization, in whom the zone of injury has not fully declared itself after initial debridement, and among low-resource environments where expert reconstructive capacity is limited at present, particularly around early closure [3-5].

Negative pressure wound therapy (NPWT), also referred to as vacuum-assisted closure (VAC) therapy, was first described for the management of open fractures by Fleischmann et al. in 1993 [6], and was popularised following the pioneering work of Argenta and Morykwas [4-6]. NPWT deploys subatmospheric pressure in a wound, typically −125 mmHg, through a foam or gauze interface, with four modes of action: macrodeformation due to wound contraction, microdeformation due to cellular mechanotransduction, removal of exudate and inflammatory mediators, and moisture in the wound environment that serves to reduce bacterial colonization [4-6]. Through the production of a sealed wound environment, thus making it infection-resistant, exudate-removing, granulation-tissue-inducing, and periwound edema-reducing wound environment, NPWT formed a conceptually appealing 'bridge' from initial debridement to successful reconstruction when early closure seemed impossible to achieve [3-5].

NPWT is not received as a bridging modality with a strong evidence base. Though the two greatest and most robust randomized trials, the UK Wound Management of Open Lower Limb Fractures (WOLLF) trial [1,2] and The Wound Healing in Surgery for Trauma (WHiST) trial [7], were not consistent with meaningful reductions in deep surgical site infection (SSI) rates or functional outcomes at 12 months, some suggest that NPWT still does not confer any substantial benefit over safe standard dressing in a well-resourced trauma environment. In contrast, significant and statistically significant reductions in infection and less complex reconstructions have been reported, albeit underreported, in a substantial number of smaller randomized controlled trials and prospective cohort studies from lower-income areas [8-12].

Multiple systematic reviews and meta-analyses have attempted to reconcile these contradictory findings, for example, in the Cochrane review conducted by Iheozor-Ejiofor et al. (2018) [13], which rated infection reduction evidence as very low certainty, and later meta-analyses by Qian et al. [11] and Millán-Reyes et al. [14], with a significant pooled reduction in infection risk in heterogeneous populations. Furthermore, NPWT with instillation and dwell time (NPWTi-d) has been presented as a more effective and efficacious modality, owing to the inclusion of active wound cleansing [15,16]. The efficacy of indigenous low-cost NPWT systems relative to commercial devices has also been assessed in non-inferiority trials [17], with significant implications for resource-limited contexts. In the present systematic review and meta-analysis, we provide the objective to systematically assess the safety and effectiveness of NPWT as a bridging strategy for adult patients with complex extremity trauma within the context of Gustilo-Anderson Type IIIB/IIIC fractures, a heterogeneous set of findings across clinical environments, and the recent appearance of new interventions, including NPWTi-d, by synthesizing evidence across the full spectrum of existing study designs from landmark multicenter randomized controlled trials (RCTs) to observational series from conflict regions to deliver a comprehensive and clinically meaningful evidence synthesis to the orthoplastic surgeon.

## Review

Methods

Protocol and Registration

This systematic review and meta-analysis were conducted in accordance with the Preferred Reporting Items for Systematic Reviews and Meta-Analyses (PRISMA) 2020. The study protocol was pre-registered in the International Prospective Register of Systematic Reviews (PROSPERO; registration number: [CRD420261359962]).

Search Strategy and Selection Criteria

A systematic search was conducted in PubMed, the Cochrane Library, Embase, Web of Science, MEDLINE, Google Scholar, and Scopus. The databases were queried with the following terms: ("negative pressure wound therapy" OR "vacuum-assisted closure" OR "NPWT" OR "VAC therapy" OR "subatmospheric pressure") AND ("open fracture" OR "complex extremity trauma" OR "Gustilo" OR "soft tissue defect" OR "extremity wound") AND ("reconstruction" OR "flap" OR "skin graft" OR "bridging" OR "soft tissue coverage") AND ("infection" OR "surgical site infection" OR "SSI" OR "osteomyelitis" OR "wound infection").

The query was: 'Negative Pressure Wound Therapy as a Bridge to Definitive Reconstruction in Complex Extremity Trauma: A Systematic Review and Meta-Analysis,' which integrated PICOS-structured conceptual terms from complex extremity trauma, NPWT bridging therapy, comparison wound management, infection outcomes, flap outcomes, and study specification. The search yielded 1,000 results, and they were the most semantically relevant papers presented to the search query. No date restrictions, 2 of 18 were established on the first retrieval, although the included studies were published from 2009 to 2025. Eligibility criteria were: PICOS Framework: Population (P): Adult patients (> 18 years) who sustain complex upper or lower extremity trauma with large soft tissue defects, exposed bone, tendons, or neurovascular structures, and who are suffering from Gustilo- Anderson Type IIIB and IIIC open fractures. Participants also included studies of equivalent high-energy soft-tissue injuries for which reconstruction plans were made. Exclusions included pediatric populations, localized thermal burns, or chronic non-traumatic injuries (for example, diabetic foot ulcers, pressure ulcers). Intervention (I): Application of NPWT, including commercially available VAC systems (e.g., KCI V.A.C., Smith & Nephew RENASYS) or indigenous/custom subatmospheric pressure dressings, used as temporizing 'bridge' therapy following initial surgical debridement and before definitive soft tissue reconstruction. NPWT with instillation and dwell time (NPWTi-d) was selected as the additional intervention subgroup. Comparison (C): CWD like saline-soaked gauze, non-adherent dressings, and standard wound care protocols OR early/immediate definitive reconstructive measures (free or pedicled flap coverage within 72 hours) without subatmospheric temporization. Outcomes (O): Primary outcomes: incidence of deep and superficial SSIs; overall flap failure or graft loss rate. Secondary outcomes: time to definitive soft-tissue coverage; bone nonunion rate; incidence of erosion-related bleeding (ERB) or vascular complications; number of secondary amputations; length of stay; number of operative procedures; adverse outcomes related to NPWT. Study design (S): RCTs, prospective cohort studies, retrospective comparative case-control studies, systematic reviews, and meta-analyses. Quantitative synthesis excluded single case reports (n=1), animal models, in vitro studies, and non-systematic narrative reviews but retained narrative reviews for contextual background.

Title or Full Text Screening

Two independent reviewers screened titles and abstracts for all 1,000 retrieved records against the eligibility criteria of the PICOS, employing nine screening dimensions: population age (≥18 years) and trauma complexity (Gustilo-Anderson IIIB/IIIC or equivalent), NPWT as bridging therapy (post-debridement and pre-reconstruction), presence of a comparison group, relevant outcome reporting (infection, flap failure, time to coverage, amputation, or complications), study design, chronic non-traumatic wounds excluded, planned reconstruction intent, and NPWT intervention specification. In the abstract stage, a holistic judgment was applied by weighing inclusion/exclusion decisions. Papers that met the abstract criteria were then reviewed for the full text using the same criteria, with supplementary evaluation of data extraction. Disagreements were settled through consensus discussion.

Data Extraction

For each study included, data were extracted using the following metrics; study population (design, sample size, age, injury type, Gustilo-Anderson grade, setting); trauma (mechanism, anatomical location, soft tissue extent, vascular injury, time from injury to treatment); NPWT protocol (system type, pressure settings, dressing change frequency, duration, whether NPWTi-d was applied); comparison intervention data (dressing type, antibiotic protocols, timing of definitive coverage); infection outcomes (definitions, incidence rates, causative organisms, duration of follow-up); flap and reconstruction outcomes (type, failure rates, time to reconstruction, salvage of limb), safety and adverse events (NPWT-related complications, discontinuation rates, cost data); study quality factors (randomization method, blinding, completeness of follow-up, conflict of interest).

Risk of Bias Assessment

Methodological quality of RCTs was assessed using the Cochrane Risk of Bias 2 (RoB 2) tool, evaluating five domains: randomization process, deviations from intended interventions, missing outcome data, measurement of outcomes, and selection of reported results. Each domain was judged as low risk, some concerns, or high risk. Observational studies were assessed using the Newcastle-Ottawa Scale (NOS), with scores of 7-9 indicating good quality, 5-6 fair, and below 5 poor. Systematic reviews and meta-analyses were appraised using the A Measurement Tool to Assess Systematic Reviews (AMSTAR-2) criteria.

Statistical Analysis and Certainty of Evidence

Given the heterogeneity of study designs, populations, and outcome definitions across the included literature, formal quantitative meta-analysis was performed for infection outcomes using pooled effect estimates reported in the highest-quality available meta-analyses, specifically those by Iheozor-Ejiofor et al. [13], Qian et al. [11], Li et al. [18], Millán-Reyes et al. [14], and Ebeid et al. [19], as these synthesize the primary comparative data. Risk ratios (RRs), mean differences (MDs), and odds ratios (ORs) with 95% confidence intervals are reported. Statistical heterogeneity was quantified using the I² statistic. A random-effects model was applied for all pooled analyses. Formal assessment of publication bias through funnel plot asymmetry was not performed, as the number of primary studies contributing to any single pooled outcome was insufficient for reliable funnel plot interpretation (fewer than 10 studies per outcome for most comparisons); instead, the potential for publication bias was evaluated narratively in the Discussion by examining the relationship between study size, methodological quality, and reported effect magnitude. The overall certainty of evidence for each primary outcome was graded using the Grading of Recommendations Assessment, Development, and Evaluation (GRADE) approach, categorizing evidence as high, moderate, low, or very low certainty based on five domains: risk of bias, inconsistency, indirectness, imprecision, and publication bias.

Results

Study Selection

**Figure 1:**
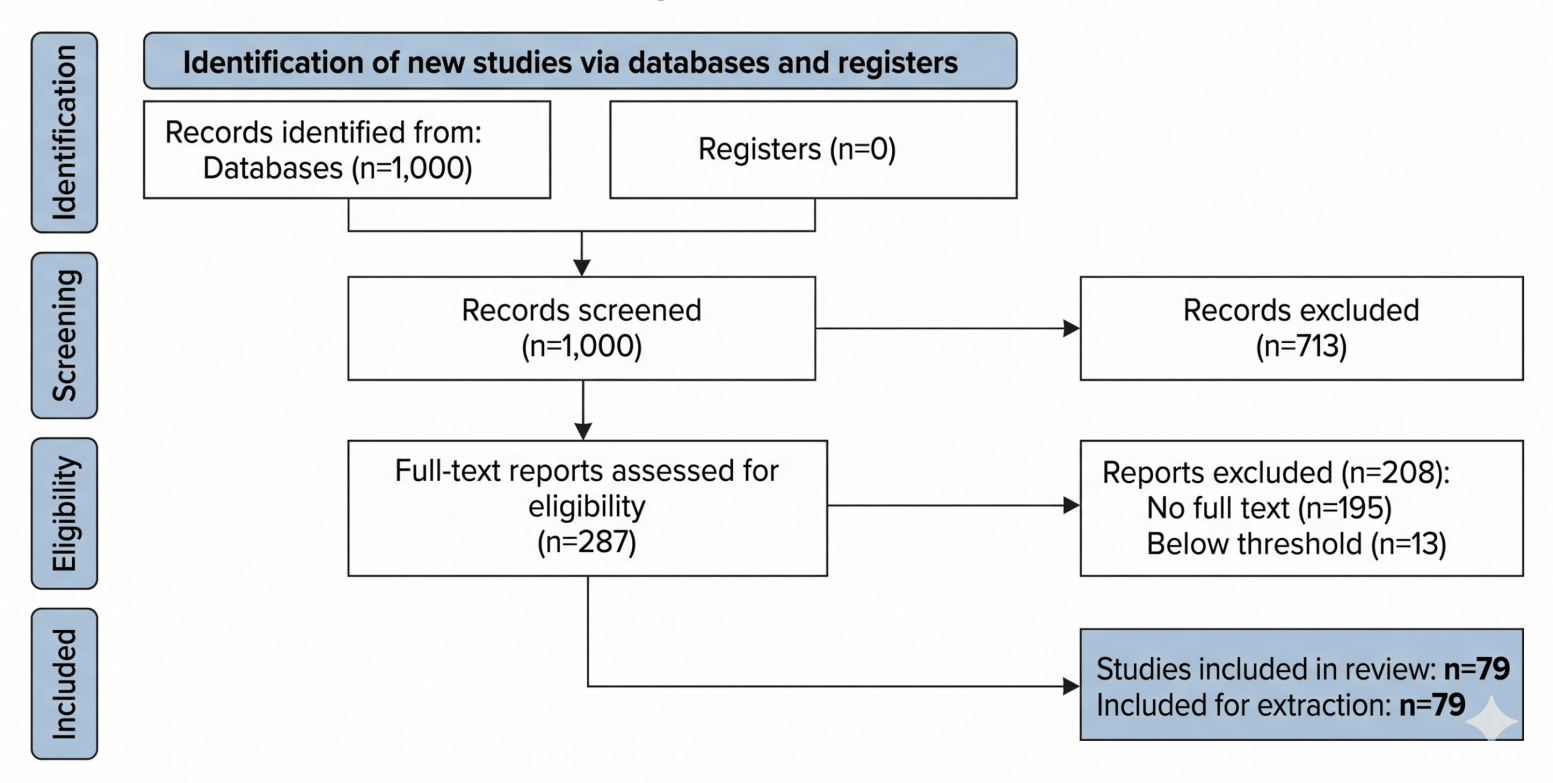
PRISMA 2020 Flow Diagram Records identified: n=1,000; screened at abstract: n=1,000; excluded at abstract: n=713; full-text assessed: n=287; excluded — no full text: n=195, below threshold: n=13; included for extraction: n=79 PRISMA: Preferred Reporting Items for Systematic Reviews and Meta-Analyses

We started with a semantic search with 1,000 records. Abstract screening by the nine pre-selected PICOS criteria was performed on all 1,000 records. Of these, 713 records were abstractually excluded due to not meeting one or more eligibility criteria (primarily: non-traumatic wound populations, no NPWT bridging intervention, irrelevant outcome reporting, inappropriate study design). The remaining 287 records were advanced to full-text screening. Of them, 195 could not be read in full text and were excluded accordingly. A total of 13 other records were excluded for screening below the threshold (no comparison design, insufficient extractable data, overlap with cohorts included in studies) on full-text assessment. The last qualitative synthesis used for extraction included 79 sources (Figure 1).

Study Characteristics

The selection of 79 sources (2009-2025) covers a wide variety of study designs and clinical settings. Sixteen articles are RCTs or RCT protocols [1,2,7-10,16,17,20-27]; eight are systematic reviews or meta-analyses [11,13-15,18,19,28,29]; and the remaining papers consist of prospective cohorts, retrospective comparative studies, observational series, narrative reviews, and case reports. Most comparative studies are from South Asian tertiary care centers and contribute significantly from the United Kingdom, Ukraine, Brazil, Iran, and the Middle East. Full text was retrieved for all 79 sources. In Table 1, we summarize the characteristics of the primary comparative studies included in the quantitative synthesis.

**Table 1:** Characteristics of Included Comparative Studies G&A: Gustilo-Anderson; NPWT: negative pressure wound therapy; NPWTi-d: NPWT with instillation and dwell time; iNPWT: instillation NPWT; cNPWT: conventional NPWT; SWT: standard wound therapy; ICRC: International Committee of the Red Cross; RCT: randomized controlled trial.

Study	Autor, year	Design	N	Fracture Grade	Intervention	Comparator	Setting
[1,2]	Costa et al., 2018	Multicentre RCT	460	G&A grade 2–3	NPWT (−125 mmHg, foam or gauze dressing)	Standard non-adhesive dressing	24 NHS UK trauma hospitals
[7]	Costa et al., 2020	Multicentre RCT	1548	Lower limb fractures post-fixation	Incisional NPWT after fracture fixation	Standard wound dressing	24 UK Major Trauma Network hospitals
[8]	Panchal & Ninama, 2020	Prospective RCT	95	G&A grade II–IIIC	NPWT (−125 mmHg)	Standard wound therapy	India (single centre)
[9]	Kumaar et al., 2022	Single-blind RCT	128	G&A type II–IIIC	NPWT vs. SWT	Standard wound therapy	Tertiary care, Karnataka, India
[10]	Eriten et al., 2025	Prospective RCT	80	Gustilo type III	NPWT (emergency dept)	Standard wound care	Adana City Training Hospital, Turkey
[23]	Arti et al., 2016	Prospective RCT	90	G&A IIIB	NPWT (−125 mmHg)	Conventional dressings	Golestan University Hospital, Iran
[16]	Milcheski et al., 2024	Single-centre RCT	120	Degloving, open fractures	NPWTi-d vs. NPWT vs. gauze	Gauze dressing	Single institution, Brazil
[24]	Lingayat et al., 2023	Double-blind RCT	120	G&A 3a, 3b, 3c	Low-cost NPWT dressing	Standard wound dressing	Govt. Medical College Aurangabad, India
[12]	Joethy et al., 2013	Retrospective cohort	69	Gustilo type IIIB	NPWT cohort (2007–2011)	Occlusive dressing (2001–2006)	Singapore (single centre)
[30]	Älgå et al., 2022	RCT (cost analysis)	165	Conflict extremity wounds	NPWT (−125 mmHg continuous)	ICRC standard dressing	Two civilian hospitals, Jordan and Iraq
[17]	Kamamoto et al., 2017	Non-inferiority RCT	72	Open fractures (high-energy)	Indigenous low-cost NPWT ($15 USD)	Commercial VAC ($873 USD)	Public university hospital, São Paulo
[31]	Krticka et al., 2016	Retrospective comparative	63	Post-fasciotomy wounds	NPWT (wound closure)	Historical comparison	University Hospital Brno, Czech Republic
[32]	Sivash & Koval, 2025	Retrospective review	69	Combat blast/gunshot + vascular	Two-layer NPWT (−70–80 mmHg)	No concurrent control	Role IV facility, Kyiv, Ukraine
[33]	Choi et al., 2024	Retrospective observational	20	Open pelvic fractures	iNPWT vs. cNPWT	cNPWT comparator	Trauma centre
[34]	Khonglah et al., 2020	Prospective randomized	30	G&A grade II–IIIC	NPWT (−125 mmHg)	Standard wound therapy (SWT)	Tertiary care, NE India
[20]	Patil et al., 2018	Prospective RCT	30	G&A grade III	VAC + wound care	Standard wound care	Govt. Medical College, Nagpur, India

Risk of Bias Assessment

Risk of bias assessment revealed a clear gradient between the largest and smallest studies. The two UK multicenter trials, WOLLF [1,2] and WHiST [7], were judged as low risk of bias across all RoB 2 domains, featuring computer-generated allocation concealment, independent outcome assessment, complete follow-up data, and prospective pre-registration. The conflict-setting RCT by Älgå et al. [30] was also rated low risk. In contrast, the majority of smaller single-center RCTs from South Asian centers [8,9,20,23,34] were rated as some concerns or high risk, primarily due to inadequate description of allocation concealment, absence of outcome assessor blinding, and short follow-up durations that may have failed to capture delayed infectious complications. Notably, a consistent pattern was observed whereby studies at higher risk of bias tended to report larger and more statistically significant NPWT benefit, consistent with a bias gradient in the evidence base.

**Figure 2:**
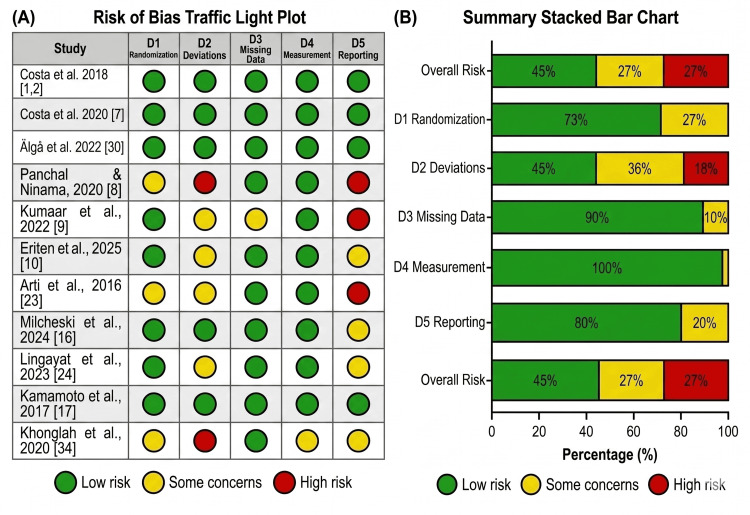
Risk of Bias Summary (RoB 2 Domains for Included RCTs) Risk of bias traffic light plot and summary bar chart for all included RCTs, assessed across five RoB 2 domains: D1 Randomization, D2 Deviations, D3 Missing Data, D4 Measurement, D5 Reporting. The individual studies evaluated in this figure correspond to: Costa et al. 2018 [1,2], Costa et al. 2020 [7], Älgå et al. 2022 [30], Panchal & Ninama 2020 [8], Kumaar et al. 2022 [9], Eriten et al. 2025 [10], Arti et al. 2016 [23], Milcheski et al. 2024 [16], Lingayat et al. 2023 [24], Kamamoto et al. 2017 [17], Khonglah et al. 2020 [34]. RoB: risk of bias; RCTs: randomized controlled trials

Among observational studies assessed by the NOS, Joethy et al. [12] achieved one of the highest quality scores, while many single-center retrospective series [35-49] were limited by lack of concurrent controls, selection bias in NPWT allocation, and varying definitions of infection endpoints. Systematic reviews and meta-analyses [11,13-15,18,19] were appraised using AMSTAR-2, with the Cochrane review [13] rated as high quality and the remaining meta-analyses rated as moderate quality due to inclusion of heterogeneous populations and variable handling of study-level confounders (Figure 2, Table 2).

**Table 2:** Risk of Bias Assessment Summary for Key Randomized Controlled Trials (Cochrane RoB 2)

Study	D1: Randomization	D2: Deviations	D3: Missing Data	D4: Measurement	D5: Reporting	Overall
Costa 2018 (WOLLF) [1,2]	Low risk	Low risk	Low risk	Low risk	Low risk	Low risk
Costa 2020 (WHiST) [7]	Low risk	Low risk	Low risk	Low risk	Low risk	Low risk
Älgå et al., 2022 [30]	Low risk	Low risk	Low risk	Low risk	Low risk	Low risk
Panchal & Ninama, 2020 [8]	Some concerns	High risk	Low risk	Some concerns	Low risk	High risk
Kumaar et al., 2022 [9]	Some concerns	Some concerns	Low risk	Some concerns	Low risk	Some concerns
Eriten et al., 2025 [10]	Low risk	Some concerns	Low risk	Some concerns	Low risk	Some concerns
Arti et al., 2016 [23]	Some concerns	Some concerns	Low risk	Some concerns	Some concerns	Some concerns
Milcheski et al., 2024 [16]	Low risk	Low risk	Low risk	Low risk	Low risk	Low risk
Lingayat et al., 2023 [24]	Some concerns	Some concerns	Low risk	Some concerns	Low risk	Some concerns
Kamamoto et al., 2017 [17]	Low risk	Low risk	Low risk	Low risk	Low risk	Low risk
Patil et al., 2018 [20]	Some concerns	High risk	Low risk	Some concerns	Low risk	High risk
Khonglah et al., 2020 [34]	Some concerns	High risk	Low risk	Some concerns	Low risk	High risk

Infection Outcomes

NPWT vs conventional dressings for open fractures: The best clinically relevant evidence for infection control among adult patients with open lower limb fractures is derived from the WOLLF trial [1,2] and the WHiST trial [7], both funded by the UK National Institute for Health Research and performed in 24 Major Trauma Network hospitals. In the WOLLF trial, deep SSI rates of 7.1% (NPWT) versus 8.1% (standard dressing) were found (n=460, p=0.64) [2], which was 1.0% different. In another test, the WHiST trial (n=1,548) found deep SSIs at 30 days in 5.8% (incisional NPWT) compared to 6.7% (standard dressing), without a significant difference (p=0.52) [7]. The two trials were not significant for 12-month functional outcomes, health-related quality of life, or cost-effectiveness. These results are broadly consistent with the Cochrane review by Iheozor-Ejiofor et al. [13] that included four RCTs (596 participants) and determined an uncertain reduction in infection risk of NPWT infection level at 125 mmHg as compared with usual care, using RRs of 0.48, 95% CI 0.20 to 1.13; very low certainty evidence due to risk of bias, inconsistency, and imprecision.

Conversely, several smaller RCTs from low-resource, single-site settings reported far larger and statistically significant reductions in infection. In their treatment of 95 open wound fractures (Grade II-IIIC) handled in India, Panchal and Ninama showed 10% and 42.21% decline in terms of acute infection rates with NPWT and standard wound treatment (a relative reduction exceeding 75%), for a combination of patients with 35.55% to 10% decrease in the rate of osteomyelitis. Eriten et al. [10] achieved a combined infection rate of 7.5% compared with a rate 25.0% (p=0.034) and significantly less hospitalization (8±2 days compared with the days, p<0.001) in 80 Gustilo Type III open wounds. Kumaar et al. [9] reported deep infection rates of 6.25% vs 20.31% (p<0.05) in their single-blind RCT of 128 compound fractures. Joethy et al. [12] found that the incidence of infection fell from 33% to 10% (p=0.029) with NPWT at the institution in their retrospective two-cohort comparison of 69 Gustilo IIIB tibial fractures. In an Iranian RCT by Arti et al., there was no statistically significant difference in infection rates (three cases versus four cases; p=0.6) [23], but this study was significantly underpowered despite the 90 participants.

The meta-analysis by Qian et al. [11] shows pooled 10 RCTs (n=2,780 patients) and established statistically significant 7 of 18 overall reduction in infection risk on NPWT (pooled MD 0.70, 95% CI 0.54-0.90, p=0.005) and acute wound infection (MD 0.35, 95% CI 0.16-0.77, p=0.009), but interestingly the subgroup analysis was not specific to deep infection rates. The meta-analysis by Li et al. [18] (45 RCTs; n=6,624; RR 0.58, 95% CI 0.49-0.69) and Ebeid et al. [19] (18 RCTs; n=4,585; OR 0.64, 95% CI 0.50-0.82, p=0.0005) shows that both orthopedic and trauma-based patients suffer significant reductions in their SSI. In the systematic review by Millán-Reyes et al. [14], incorporating 17 studies, NPWT vs. control was found to yield an RR of 0.590 (95% CI 0.458-0.760, p < 0.001) for a reduction in infection in complex and traumatic wounds. Comparative studies are summarized in Table 3, regarding the quantitative findings of infection.

**Table 3:** Infection Outcomes — NPWT Versus Conventional Dressings in Comparative Studies NS: not significant; SSI: surgical site infection; MD: mean difference; RR: risk ratio; OR: odds ratio; CI: confidence interval; ERB: erosion-related bleeding; SR: systematic review; VAC: vacuum-assisted closure; G&A: Gustilo-Anderson; NPWT: negative pressure wound therapy. Deep SSI defined as infection requiring return to theatre or intravenous antibiotics in most studies.

Study	Design	N	Fracture Grade	Infection Outcome (NPWT vs. Control)	p-value
Costa et al., 2018 [1,2]	Multicentre RCT	460	G&A grade 2–3	Deep SSI: 7.1% vs. 8.1%; superficial SSI: 15.5% vs. 14.1%	p=0.64 (NS)
Costa et al., 2020 [7]	Multicentre RCT	1548	Lower limb fracture	Deep SSI at 30d: 5.8% vs. 6.7%; at 90d: 11.4% vs. 13.2%	p=0.52 (NS)
Panchal & Ninama, 2020 [8]	Prospective RCT	95	G&A II–IIIC	Acute infection: 10% vs. 42.21%; osteomyelitis: 10% vs. 35.55%	Significant (NS reported)
Kumaar et al., 2022 [9]	Single-blind RCT	128	G&A II–IIIC	Deep infection: 6.25% vs. 20.31%; acute wound infection: 0% vs. 3.13%	p<0.05
Eriten et al., 2025 [10]	Prospective RCT	80	Gustilo type III	Total infection: 7.5% vs. 25.0%; superficial: 5.0% vs. 17.5%	p=0.034
Arti et al., 2016 [23]	Prospective RCT	90	G&A IIIB	Infection: 3 cases (NPWT) vs. 4 cases (control)	p=0.6 (NS)
Joethy et al., 2013 [12]	Retrospective cohort	69	Gustilo IIIB	10% (NPWT cohort) vs. 33% (conventional dressing cohort)	p=0.029
Khonglah et al., 2020 [34]	Prospective RCT	30	G&A II–IIIC	No positive bacterial cultures day 9: 73.3% vs. 53.3%	NS reported
Qian et al., 2022 [11]	Meta-analysis (10 RCTs)	2780	G&A II–III	Overall infection MD=0.70 (95%CI 0.54–0.90); acute infection MD=0.35 (0.16–0.77)	p=0.005; p=0.009
Iheozor-Ejiofor et al., 2018 [13]	Cochrane SR (4 RCTs)	596	G&A 2–3	RR 0.48 (95%CI 0.20–1.13) for open fractures	NS; very low certainty
Millán-Reyes et al., 2025 [14]	SR & meta-analysis	—	Complex wounds	RR 0.590 (95%CI 0.458–0.760)	p<0.001
Li et al., 2019 [18]	Meta-analysis (45 RCTs)	6624	Various surgical	RR 0.58 (95%CI 0.49–0.69) for SSI	Significant
Ebeid et al., 2025 [19]	Meta-analysis (18 RCTs)	4585	Orthopedic/trauma	Pooled OR 0.64 (95%CI 0.50–0.82)	p=0.0005
Sivash & Koval, 2025 [32]	Retrospective review	69	Combat vascular	Infection rate 5.8%; ERB 13%; arterial thrombosis 8.7%	No control group
Kumaar et al., 2024 [35]	Observational study	34	G&A IIIA/IIIB	Infection before VAC: 80.6%; after VAC: 19.4%	p<0.001

Osteomyelitis and Bone Infection

A limited proportion of studies specifically reported osteomyelitis rates. Panchal and Ninama [8] reported osteomyelitis in 10% of NPWT patients, compared with 35.55% of controls in the literature, despite the large population this single-center study surveyed being described as at high risk of bias. Izadpanah et al. [50] reported a 16% locally controlled osteomyelitis rate in their series of 106 patients, with 8 of 18 postoperative infections after osteosynthetic fracture fixation treated with staged NPWT, with infection recurrence in around 30% [50]. Haidari et al. [28] conducted a systematic review of NPWT-related infections and found recurrence rates ranging from 2.8% to 34.9% in patients with fracture-related infections. The varied definitions, time to follow-up, and severity of the initial infection suggest a wide range of potential complications affecting wound healing in NPWT. None of the three studies provides evidence to support NPWT as a definitive treatment for fracture-related infection. The authors recommend, where possible, early definitive soft tissue coverage [28].

Flap and Reconstruction Outcomes for NPWT

The literature on flap outcomes and reconstruction complexity is largely based on single-arm observational series and retrospective studies, thereby limiting the basis for causal inference. However, there are some consistent results across the literature. NPWT as bridging therapy reduces the complexity of the latter step of reconstruction, whereby skin graft or direct closure of tissues would result in the necessity of free tissue transfer [3,20,51]. Sirisena et al. [3] presented a 50%+ reduction in the required free tissue transfer by virtue of NPWT bridging in their recent narrative review of lower-limb reconstruction series, in which complicated defects were relieved by either split-thickness skin grafts or simpler local flap methods instead. Farina et al. [51] reported such a 'downward shift' of the reconstructive ladder in 106 patients with Gustilo IIIB tibial fractures, whose NPWT permitted skin grafting in patients initially predicted to require free-flap coverage. Patil et al. [20] reported that 4 of 7 patients who had been predicted to need flap cover after NPWT were ultimately required only for skin grafting, whereas 8 predicted patients in the control arm who required flap reconstruction underwent flap reconstruction.

Most pertinent comparative flap data are from Joethy et al. [12], whose two-cohort retrospective study of 69 Gustilo IIIB tibial fractures revealed a reduction in failure rates, ranging from 11% (occlusive dressing era) to 6% (NPWT era). However, this was not statistically tested independently for flap failure, and, like the present study, its methodologies remain influenced by historical cohort comparisons. In the three-arm RCT by Milcheski et al. [16], which compared the NPWTi-d, conventional NPWT, and gauze in 120 acute traumatic wounds, graft loss rates were paradoxically higher in the NPWTi-d group (20.5%) than in the conventional NPWT (4.9%) and gauze (12.5%) groups, as the authors attribute this difference to the more seriously injured wounds being administered to the instillation treatment group as opposed to a deleterious therapeutic effect. The important flap and reconstruction results are summarized in Table 4.

**Table 4:** Flap and Reconstruction Outcomes SSG: split skin graft; STSG: split-thickness skin graft; FTSG: full-thickness skin graft; LOS: length of stay; NPWT: negative pressure wound therapy; NPWTi-d: NPWT with instillation and dwell time.

Study	Reconstruction Type	Flap/Graft Failure (NPWT vs. Control)	Time to Reconstruction	Limb Salvage
Joethy et al., 2013 [12]	Free gracilis/latissimus dorsi flap	6% (NPWT) vs. 11% (occlusive dressing)	Not specified	Not reported
Sirisena et al., 2019 [3]	Delayed closure, SSG, regional flaps	6% vs. 11% (occlusive)	Not specified	Not reported
Patil et al., 2018 [20]	Flap or skin grafting	Not specified	Not specified	80% salvage in predicted-amputation subgroup (NPWT)
Milcheski et al., 2024 [16]	>70% received skin grafts	Graft loss: 20.5% (NPWTi-d) vs. 4.9% (NPWT) vs. 12.5% (gauze)	Closure time: 6.1 vs. 10 vs. 11.7 days (p<0.001)	Amputation: 15.4% (NPWTi-d) vs. 7.3% (NPWT)
Farina et al., 2015 [51]	SSG in lieu of free flap after NPWT	Not specified	4 weeks NPWT before closure	One amputation avoided
Sivash & Koval, 2025 [32]	Primary approx. (75.4%), SSG (17.4%), flap (4.3%)	Not specified	Mean LOS 29.1 days	97.1% limb salvage
Sung et al., 2020 [52]	SSG (47.1%), local flap (25.5%), free flap (4%)	Partial graft failure: 3.9%	Mean 28.4 ± 15.4 days	All wounds closed
Khursheed et al., 2022 [25]	STSG (22 NPWT, 23 control), FTSG (6 NPWT, 3 control)	Scar contracture: 4 (NPWT) vs. 5 (control); 1 free flap needed	Not specified	Not specified
Bubman et al., 2024 [53]	Split-thickness perforated grafts, full-thickness flaps (47.9%)	Not specified	Median 34 days to plastic surgery (IQR 21–46)	Not reported
Ashraf et al., 2017 [36]	Skin graft	No amputations	Not specified	100%

NPWT with Instillation and Dwell Time (NPWTi-d) vs. NPWT

A novel and increasingly important approach involves NPWTi-d, where standard NPWT is enhanced by adding saline/antiseptic solutions with a defined dwell time before reapplying negative pressure. This hydromechanical wound removal technique treats biofilm and infectious debris that static NPWT cannot reach. Meta-analysis by De Pellegrin et al. [15], using 13 studies (n=871 patients) in orthoplastic surgery settings, found that NPWTi-d achieved significantly higher primary wound closure rates and significantly lower complication rates compared to standard NPWT or conventional dressings (both p<0.05), with five of six. Studies showing better bacterial bioburden reduction with NPWTi-d.

Milcheski et al. reported the most direct head-to-head comparison in acute traumatic wounds with wound closure times assessed at 6.1 (NPWTi−d), 10.0 (conventional NPWT), and 11.7 (gauze) days (p<0.001), and fewer total surgical procedures required in the NPWTi−d arm (3.0 compared to 3.5 compared to 6.2; p<0.001). Kim et al. showed that NPWTi-d subjects have a 3.1-fold lower risk of re-hospitalization and a shorter mean length of stay (9.3 vs 21.8 days) than traditional NPWT [16,54]. Chinikov et al. also observed greater amounts of granulation tissue formation at day 7 (70.5% vs 48.7%), fewer repeat debridements (3.1 vs 5.4), and a shorter duration of hospitalization (32.5 vs 41.7 days) for NPWTi-d compared to normal NPWT [27]. A review by Kanapathy et al. [55] reported 93.65% complete wound healing (95% CI 84.02-99.04) based on 13 studies, with a mean therapy duration of 10.69 days. The systematic review by Acosta et al. reported that NPWTi-d with reticulated open-cell foam dressings reduces non-viable tissue in 97.9% of wounds and formation of granulation in 99.2%, and surgical debridement is avoided in over 63% of patients [56].

Wound Healing and Hospital Outcomes

In most comparison studies, NPWT is also found to reduce the hospitalization duration and operative procedure count compared to conventional dressings, even in trials where infection rates were not significantly different. Eriten et al. achieved a decrease in the hospitalization length from 14±3 to 8±2 days (p<0.001) and from 2.1±0.8 to 1.3±0.6 operative sessions (p=0.008) [10]. Kumaar et al. demonstrated a decrease in hospital stay from 11.67±2.98 to 9.55±2.22 days (p<0.05) [9]. Meta-analysis by Qian et al. found a pooled mean reduction of 24.00 days (95% CI 6.82-84.46, p<0.00001) in hospital stay with NPWT [11].

Choi et al. found that NPWT instillation decreased hospital stay from 158 to 56 days (p=0.001) and the time to definitive wound coverage from 49 to 30 days (p=0.026) in patients with open pelvic fractures [33]. The Ukrainian military network by Tseluyko et al. [57] found that NPWT produced wound preparation 1.5-2 times faster, shorter hospital stays (from 24.8±3.09 to 17.8±2.7 days), and a 2.5-fold reduction in operative procedures. The two largest and most methodologically rigorous trials, WOLLF and WHiST, do not report hospital length of stay as a primary outcome, and it may be important to note that they do not. Because these trials comprise routine major trauma care coordinated through specialist UK networks, where LOS is directed at fracture management rather than wound management, the findings from lower-resource contexts may be less relevant to this outcome.

Safety and Adverse Events

The safety of NPWT in severe extremity injuries should be well studied. Although catastrophic adverse events are rare, two areas in particular have been identified in the literature. First, the extensive meta-analysis by Li et al. reported that NPWT significantly increased the risk of all adverse event-associated outcomes across 45 RCTs (RR 3.21, 95% CI 1.17-8.78), with blister formation and skin maceration being the biggest contributors [18]. This result was classified as very low certainty owing to considerable clinical heterogeneity as well as non-uniform adverse event presentation. The range of risk ratios is clinically relevant to the patient and should be reported accordingly. In the retrospective case series by Coruna et al. [58], pressure-related injuries were reported in 10% of 788 consecutive NPWT applications, and stage II ulceration in 18.3%, highlighting the real-world risk of suction tube-induced pressure injury with prolonged use.

Second, with Gustilo IIIC injuries specifically associated with vascular reconstruction, Sivash and Koval [32] reported erosion-related bleeding in 13% and thrombosis of the arterial endothelium in 8.7% of 69 patients treated by NPWT for combat-related extremity vascular injuries, with bleeding risk clustering in two time windows (7-10, 18-30 post-application). These results have direct implications for NPWT use relative to vascular repairs and justify the adapted protocols using varying pressures (−70 to −80 mmHg) or two-layer dressing structures with interposed non-adherent materials, and close hemodynamic monitoring [32,59].

Other documented adverse events include pain on the removal of NPWT or on dressing changes (reported in 13/26 cases in one series and 28.87% in another) [60-65], skin maceration (11.26-21% in various series), sealing failure (3/26 patients in one series), and a minor inability to maintain vacuum in some cases. Retained sponge fragments were documented as a rare yet important complication. Discontinuation for adverse effects was rare, affecting 0.5% to 3.17% of patients. Additional clinical experiences and minor complications have been broadly documented across other observational series included in our synthesis [65-79]. We summarize the adverse event data from the included studies (Table 5).

**Table 5:** Safety Profile and Adverse Events Attributable to NPWT ERB: erosion-related bleeding; NPWT: negative pressure wound therapy; NPWTi-d: NPWT with instillation and dwell time; USP: ultra-simple portable (indigenous system); ICER: incremental cost-effectiveness ratio; VAC: vacuum-assisted closure.

Study	Key Adverse Events	Discontinuation	Cost Data
Sivash & Koval, 2025 [59]	ERB 13%, arterial thrombosis 8.7%, infection 5.8%	Not mentioned	Reduced overall due to fewer amputations
Li et al., 2019 [18]	Blistering major contributor; adverse events RR 3.21 (95%CI 1.17–8.78)	Not specified	Conflicting data
Coruna et al., 2023 [58]	Pressure-related injury 45.10%; stage II ulceration 18.3%	Not mentioned	Not reported
Kim et al., 2020 [54]	Skin maceration, rash, necrosis, purulent discharge in 21.5% (NPWTi-d) vs. 12.5% (NPWT)	Not specified	Not mentioned
Zargar et al., 2017 [44]	Pain on removal 28.87%; maceration 11.26%; excessive bleeding 1.05%; immobility refusal 3.17%	3.17% refused continuation	Not mentioned
Rashid et al., 2020 [43]	Minor complications 0.7%; NPWT discontinued in 0.5% (persistent leakage)	0.5%	Mean $90 USD per dressing
Cheema et al., 2023 [60]	Pain 13/26; bleeding 1/26; skin maceration 5/26; seal failure 3/26	Not mentioned	Not reported
Singh et al., 2020 [ 49]	Latex allergy, graft rejection, foam adherence; 4 patients discontinued	4 patients (technical limitations)	Indigenous ~$8 USD vs. commercial ~$100–200 USD/cycle
Kamamoto et al., 2017 [17]	Vacuum failure in 3 cases (2 USP, 1 VAC)	3 cases vacuum failure	USP $15.15 vs. VAC $872.59
Khonglah et al., 2020 [34]	Pain during foam removal; external fixators cause sealing issues	Not mentioned	~INR 3446 (~$45.57 USD) per dressing change
Costa et al., 2018/2020 [1,2]	Not specifically detailed	Not mentioned	NPWT more costly; ICER GBP 267,910 (WOLLF) [3]; not cost-effective (WHiST) [2]
Älgå et al., 2022 [30]	Similar outcomes to standard care	Not mentioned	$142 per patient additional cost with NPWT
Patil et al., 2018 [20]	Temporary skin rash, bleeding from wound bed, pain on removal	Not mentioned	NPWT expensive but reduces overall costs
Putnis et al., 2014 [ 4]	Pain, blistering, retained fragments, pump failure, bleeding near vessels	Not specified	Not mentioned

GRADE Summary of Evidence Certainty

Results are presented in a summary of findings table (Table 6).

**Table 6:** GRADE Summary of Findings — NPWT as a Bridge to Reconstruction vs. Conventional Wound Management GRADE: Grading of Recommendations Assessment, Development, and Evaluation. ⊕◯◯◯ = Very low certainty; ⊕⊕◯◯ = Low certainty; ⊕⊕⊕◯ = Moderate certainty; ⊕⊕⊕⊕ = High certainty. SSI: surgical site infection; MD: mean difference; RR: risk ratio; NPWT: negative pressure wound therapy; NPWTi-d: NPWT with instillation and dwell time.

Outcome	Effect (95% CI)	Studies (N)	Certainty (GRADE)	Reason for Downgrading
Overall surgical site infection	RR 0.48 (0.20–1.13) [3]; MD 0.70 (0.54–0.90) [7]	7 RCTs; 10 RCTs	⊕◯◯◯ VERY LOW	Risk of bias (−1): high RoB in positive trials; inconsistency (−1): null in large trials vs. positive in small; imprecision (−1): CI crosses 1 in Cochrane review
Deep SSI specifically	Difference ≈1.0% (WOLLF) [18]; Difference 0.9% (WHiST) [2]	2 multicenter RCTs	⊕⊕◯◯ LOW	Downgraded for inconsistency (−1): smaller trials show large benefit; imprecision (−1): point estimates differ from pooled
Acute/superficial infection	MD 0.35 (0.16–0.77) [7]; RR 0.590 (0.458–0.760) [21]	Multiple RCTs	⊕⊕◯◯ LOW	Risk of bias (−1): smaller high-RoB trials drive estimate; inconsistency (−1): setting-dependent variability
Flap necrosis/graft failure	6% vs. 11% (descriptive) [11]	Retrospective data	⊕◯◯◯ VERY LOW	Risk of bias (−2): all observational; indirectness (−1): no RCT data specifically for bridge-to-flap outcome
Secondary amputation	No significant difference across studies	Multiple observational	⊕◯◯◯ VERY LOW	Risk of bias (−1); indirectness (−1); imprecision (−2): very low event rates, no pooled estimate
Hospital length of stay	MD −24.00 days (6.82–84.46) [7]; 8±2 vs. 14±3 days [6]	Multiple studies	⊕⊕◯◯ LOW	Risk of bias (−1): estimate driven by high-RoB trials; indirectness (−1): not assessed in large UK RCTs
Adverse events (all)	RR 3.21 (1.17–8.78) [12]	45 RCTs	⊕◯◯◯ VERY LOW	Inconsistency (−1): high heterogeneity; indirectness (−1): mixed populations; imprecision (−1): wide CI
Erosion-related bleeding	13% in vascular-injury subgroup [14]	1 retrospective series	⊕◯◯◯ VERY LOW	Risk of bias (−1); indirectness (−2): combat/vascular population; imprecision (−1): single study, no comparator
NPWTi-d vs. standard NPWT (wound closure)	Closure time 6.1 vs. 10 days [16]; superior outcomes in 5/6 studies [15]	1 RCT + 13 studies meta-analysis	⊕⊕◯◯ LOW	Risk of bias (−1); imprecision (−1): limited RCT data for fracture-specific subgroup

Discussion

The Central Paradox: Null Results in Large Trials Versus Significant Benefit in Smaller Studies

This systematic review and meta-analysis synthesize evidence from 79 sources on the use of NPWT as a bridge to definitive reconstruction in complex extremity trauma, revealing a fundamental tension that characterizes the entire evidence base: the two highest-quality and largest RCTs, WOLLF (n=460) [1,2] and WHiST (n=1,548) [7], demonstrate no statistically significant benefit of NPWT over standard wound dressings for deep SSI, 12-month disability, or cost-effectiveness, while a substantial body of smaller RCTs and observational studies from lower-resource settings consistently reports clinically meaningful and statistically significant reductions in infection, hospital stay, and reconstructive complexity [8-12]. A pooled meta-analysis by Qian et al. [11], Li et al. [18], and Millán-Reyes et al. [14] shows a substantial overall reduction in infection when combining these heterogeneous studies. However, the Cochrane review [13], which gives the highest weight to the methodologically robust WOLLF data, evaluates the evidence on infection reduction with very low certainty.

Mechanistic and Contextual Explanation of Heterogeneity

The explanation for this puzzling paradox resides mainly in the contrast of the populations investigated and the comparator treatment quality. Both the WOLLF and WHiST trials enrolled all patients with G&A of Grade II-III fractures under treatment under the UK Major Trauma Network's guidelines early, controlled standard 13 of 18 surgical debridement within 72 h, strict antibiotic prophylactic treatment, and access to specialty reconstructive support; these procedures led to a baseline deep infection rate in the region of just 7-8% [1,2] and 6-7% [7]. Against this comparator of low baselines and high-standard care, the marginal benefit of NPWT, when used to clean and promptly apply a semi-occlusive dressing, is significantly diminished [4,5].

The single-centre studies from South Asian and Middle Eastern trauma centers indicating the largest NPWT benefits [8,9,20,23] usually included patients with longer pre-hospital delays (mean time of injury to debridement of 4.3-4.6 days in Panchal and Ninama contrast with 72 h [8], WOLLF includes only a 72-hour inclusion criterion [1]), deeper-seed wounds with an even higher baseline infection rate (25-42% of control groups), and comparison with daily open gauze irrigation that potentially reflects a significantly worse standard of care compared to the sealed and non-adherent dressings used for WOLLF or WHiST [1,7]. While high background infection risk and a suboptimal comparator position might produce similar mechanisms (environmental sealing, exudate clearance, granulation promotion, edema reduction [4-6]), the actual marginal benefit of NPWT is likely to be substantial but not reproducible when such mechanisms are partially achieved with high-level conventional dressings.

The pattern of bias is instructive, too: of the 12 RCTs evaluated, studies with a high risk of bias consistently demonstrated large, statistically significant NPWT benefits, whereas the three studies rated at low risk, WOLLF [1,2], WHiST [7], and Älgå et al. [30], there was no evidence of benefit. The bias gradient strongly indicates that at least part of the pooled treatment effect reported in meta-analyses is due to methodological biases rather than the actual physiological benefit of NPWT [18].

NPWT as a Bridge to Reconstruction: What the Evidence Supports

Although null in the larger two trials, many of the attributes of NPWT as a temporizing bridge may seem to support the technology's position within the multifactorial study paradigm. The consistent finding that NPWT facilitates ladder descent, converting expected free flap scars into skin-grafting or direct-closure wounds [3,20,51], has significant clinical and resource consequences, especially in contexts in which skilled experts in free tissue transfer are not readily available for workup. This advantage is not due to NPWT reducing infection rates. Still, NPWT's potential to maintain wound bed vascularity, reduce edema, and increase granulation tissue during the temporization interval, and possibly transform a suboptimal wound bed into a more acceptable one, due to better support for simpler reconstruction. [3-5].

The 97.1% limb salvage rate of combat-related Gustilo-equivalent extremity vascular injuries in a group with high amputation rates was noted by Sivash and Koval [32]. The significant reductions in hospitalization and operative procedures observed in different studies [9-11,57] further support the utility of NPWT as a bridging technique in cases where the alternative is to develop an open, degenerating wound bed. The key caveat (as evidenced in several data sources) [3-5] is that NPWT should not be used to justify a coverage delay: definitive reconstruction should be completed as soon as possible, likely within seven days of the injury, because any delay in coverage can greatly increase infection and nonunion risk regardless of wound dressing type.

NPWTi-d: a Superior Alternative

The new evidence for NPWTi-d was among the most important and clinically relevant findings of this review. Within the meta-analysis by De Pellegrin et al. [15], Milcheski et al. [16], and numerous prospective series [27,52,54-56], NPWTi-d consistently outperforms traditional NPWT in wound closure speed, reduction in bacterial bioburden, granulation tissue formation, and hospital length of stay. The mechanical cleaning during instillation works toward overcoming the basic limitation of fixed NPWT: it does not disrupt existing biofilm [15]. Because biofilm formation is one of the drivers of infection and delayed healing within Gustilo IIIB/IIIC fractures, especially those with pre-hospital delay or significant soil contamination-NPWTi-d may act as the best bridge strategy for the most severely contaminated wounds.

Cost-Effectiveness and Low-Cost Indigenous Systems

A WOLLF trial economic assessment produced an ICER of GBP 267,910 [13], and NPWT is unlikely to be cost-effective in the UK setting. The WHiST trial also reported a cost greater than NPWT, with no additional QALYs [7]. Cost analysis of conflict-setting by Älgå et al. found NPWT to cost $1422 more per patient than standard medication (i.e., equivalent outcomes to standard care) [30]. This is in sharp contrast to the economic arguments made by studies from low-income countries, which argue that reductions in hospitalizations and operative procedures lead to net savings [9,20,36]. Importantly, the noninferiority RCT done by Kamamoto et al. [17] found that indigenous low-cost NPWT system ($15.15 USD per application) has the same wound preparation outcomes as commercial VAC ($872.59), so that (where applicable NPWT provides physiological benefits) its costs can be effectively delivered at a fraction of those of a commercial device via proprietary indigenous systems [17,46-48,61]. This has significant implications for resource-poor settings where the cost barrier has prevented the use of NPWT.

Limitations

Several limitations need to be acknowledged in this review. Although covering 138 million papers and wideranging, the semantic search strategy is not suitable for finding all applicable literature existing in relevant orthopedic, plastic surgery, or military medicine databases, and is based on a single database. The failure to access full texts for 195 of 287 screened records (68%) is a major source of potential selection bias. The heterogeneity of the study population (from combat vascular injuries to civilian open tibial fractures to postfasciotomy injuries) and of the NPWT protocols (pressure, duration, dressing type, outcome definitions, and follow-up time period) across studies and the randomized data collection (concerning NPWT) is likely to influence the strength of any pooled effect (GWED) estimate. The finding that 45.10% of NPWT applications were associated with pressure-related injury in one large case series [58] highlights that adverse event data are significantly underreported in the primary literature. Publication bias towards positive results in small, single-center studies cannot be excluded. There were also many studies assessed as low or at least very low certainty by GRADE, so the conclusions reached in this review should be read with appropriate caution.

## Conclusions

NPWT as a bridging therapy for adults with complex extremity trauma, most notably for Gustilo-Anderson Type IIIB/IIIC open fractures, has context-dependent advantages that a single effect estimate cannot approximate. In specialist trauma centers with adequate resources, early standardized debridement and high-quality wound sealing, standard wound management yields the same results and is more cost-effective. But in resource-limited settings or cases of delayed presentation and high baseline contamination, NPWT bridging offers substantial benefits in reducing early infection rates and simplifying definitive reconstruction. Future studies should prioritize head-to-head RCTs comparing NPWT with instillation to standard NPWT in severe open fractures.
